# Treatment of increased intracranial pressure secondary to otitic hydrocephalus

**DOI:** 10.3389/fped.2026.1734335

**Published:** 2026-03-03

**Authors:** Esha Prakash, Tais Estrela, Gena Heidary, Caroline D. Robson, Eliot Shearer, Ross O’Shea, Hae-Young Kim, Alfred Pokmeng See, Ryan Gise

**Affiliations:** 1 Department of Ophthalmology, Boston Children’s Hospital, Harvard Medical School, Boston, MA, United States; 2 Department of Radiology, Boston Children’s Hospital, Harvard Medical School, Boston, MA, United States; 3 Department of Otolaryngology, Boston Children’s Hospital, Harvard Medical School, Boston, MA, United States; 4 Department of Neurosurgery, Boston Children’s Hospital, Harvard Medical School, Boston, MA, United States

**Keywords:** mastoiditis, ophthalmology, otitic hydrocephalus, papilledema, pediatrics

## Abstract

**Objectives:**

Otitic hydrocephalus is defined as increased intracranial pressure secondary to acute mastoiditis with cerebral venous thrombosis, leading to visual morbidity from papilledema. As no standardized ophthalmological treatment for papilledema exists, this study aims to evaluate the impact of corticosteroids on visual and clinical outcomes.

**Methods:**

This retrospective chart review includes children ≤18 years old, with otitic hydrocephalus and papilledema. Patients were evaluated by ophthalmology between July 2022 and July 2024 at a quaternary children's hospital. Data from ophthalmologic visits, neuroimaging, and clinical courses were recorded.

**Results:**

Fourteen patients [nine male (64%) and five female] were identified; their average age was 5.6 years (range 2–9). All were treated with acetazolamide, anticoagulation, and antibiotics. Two patients (14%) underwent otologic surgery and neurosurgery. Twelve patients received corticosteroids; four (29%) needed further neurosurgery, while eight (64%) improved with corticosteroids alone. Visual acuity (VA), Frisen grade at presentation, and time to corticosteroid initiation were not predictive of corticosteroid success. Patients who had corticosteroids only vs. those who had surgery had a reduced length of stay. Five patients who presented with retinal changes on optical coherence tomography developed signs of optic disc atrophy at final appointment.

**Conclusion:**

This series shows that there could be a potential role for systemic corticosteroids in reducing the hospital duration of patients with otitic hydrocephalus. It demonstrates the need for close follow-up, as corticosteroid success is difficult to predict. Patients who present with retinal changes may be at higher risk for optic disc atrophy, potentially affecting visual function and requiring more frequent follow-up.

## Introduction

Otitic hydrocephalus is a rare intracranial complication of suppurative otitis media and associated mastoiditis (1). It is defined as raised intracranial pressure (ICP) secondary to the middle ear or mastoid disease without radiologic evidence of ventriculomegaly (1–4). These patients can present with papilledema (optic nerve head edema secondary to raised ICP), which can lead to permanent vision loss (3, 5). Increased ICP is thought to occur because of thrombosis of the dural venous sinuses and/or internal jugular veins, leading to a rise in cerebral venous pressure and subsequent increase in central serous fluid pressure (1). It has also been proposed that there is an inflammatory component to all cerebral venous sinus thromboses (CVST) that may affect systemic and visual outcomes (6). Previous literature suggests that 15%–22% of patients with mastoiditis develop intracranial complications despite medical treatment (7, 8).

Diagnosis and management involves a multidisciplinary team, including infectious disease, otolaryngology, neurosurgery, neuroradiology, neurology, ophthalmology, and hematology (2, 3, 9, 10). Medical treatment often includes anticoagulation, antibiotics, and acetazolamide (3, 11). Surgical interventions can include otologic surgeries such as mastoidectomy and myringotomy tubes to address the primary source of infection (7, 12). Furthermore, ICP-reducing neurosurgical procedures such as ventriculoperitoneal (VP) shunt and endovascular thrombectomy may be required to be done to prevent vision loss (13).

In CVST, two studies showed that there was significant visual morbidity in both children and adults (4, 14). Both studies found that papilledema commonly progressed after diagnosis and the visual morbidity presented as an optic atrophy in children and quantifiable visual field defects in adults. The visual outcomes following otitic hydrocephalus have not been widely reported, but one recent study reported some initial visual outcomes on a small cohort (15). In that cohort, individuals required further neurosurgery because of a progression of papilledema despite the fact that the initiation of corticosteroids had an overall positive impact on vision (15).

The present study aims to provide a detailed evaluation of interventions taken for patients presenting with otitic hydrocephalus, and their efficacy and visual outcomes.

## Methods

This was a retrospective study of patients diagnosed with otitic hydrocephalus secondary to mastoiditis at a quaternary academic hospital between July 2022 and July 2024. The institutional review board approved the research protocol with a waiver of consent, given the retrospective nature of this study. The methods complied with Health Insurance Portability and Accountability Act regulations and adhered to the tenets set forth by the Declaration of Helsinki.

Pediatric patients were identified by ICD 10 codes and then a manual review was performed to confirm the diagnosis. Patients <18 years were included. Data were collected on presentation and demographics, ophthalmology examination, neurologic imaging, and medical/surgical management. Best-corrected visual acuity (BCVA), color vision, degree of papilledema as defined by Frisen grade (16), presence of optic disc atrophy, and oculomotor palsies were measured at initial and final visits by a qualified ophthalmologist. Ganglion cell layer (GCL) volume, retinal nerve fiber layer (RNFL) thickness, and retinal findings were recorded using optical coherence tomography (OCT) (Heidelberg) at initial and final visits. OCT testing is a well-established, non-invasive evaluation that can be performed in the office on awake and compliant patients. It utilizes light to create a map of the retinal tissue and can be used to identify and quantify papilledema. It can also serve as a marker of visual function and permanent visual damage.

Time to treatment was recorded as the time between initial ophthalmic review until the date of treatment initiation. The Frisen grading scale, which is a numerical scale that ranges from Grade 0 to Grade 5, was used specifically to describe optic nerve edema secondary to increased ICP or papilledema. Improvement in papilledema was defined as a decrease of at least one Frisen grade, while progression was defined as an increase of at least one Frisen grade. Time to improvement/progression of papilledema was measured from the first ophthalmic review to the date of documentation of change in papilledema. The total duration of hospital stay was measured as the number of days that the patient was admitted in hospital. If a patient was readmitted, the number of days continued from the previous admission. The other parameters measured were the opening pressure of initial lumbar puncture (LP), the peak erythrocyte sedimentation rate (ESR), white blood cell (WBC) count, C-reactive protein (CRP) level, and procalcitonin level. When available, reliable visual field (VF) testing in the form of Goldmann kinetic perimetry (Haag-Streit) and Humphrey static perimetry (Carl Zeiss Meditec) was performed. Reliable VF testing was defined as in previous pediatric studies (17). The qualitative assessment of VF testing for field defects was performed by a physician (RG) trained in neuro-ophthalmology.

Imaging consisted of computed tomography (CT), brain magnetic resonance (MR) imaging, and MR venography (MRV). CT tests included high-resolution thin section images of the temporal bones with intravenous contrast. MR images were obtained on a Siemens 3T system with multiplanar sequences of the brain and high-resolution sequences of the temporal bones without and with intravenous gadolinium. All patients underwent MR venography. Imaging was reviewed by an experienced pediatric neuroradiologist.

**Figure 1:**
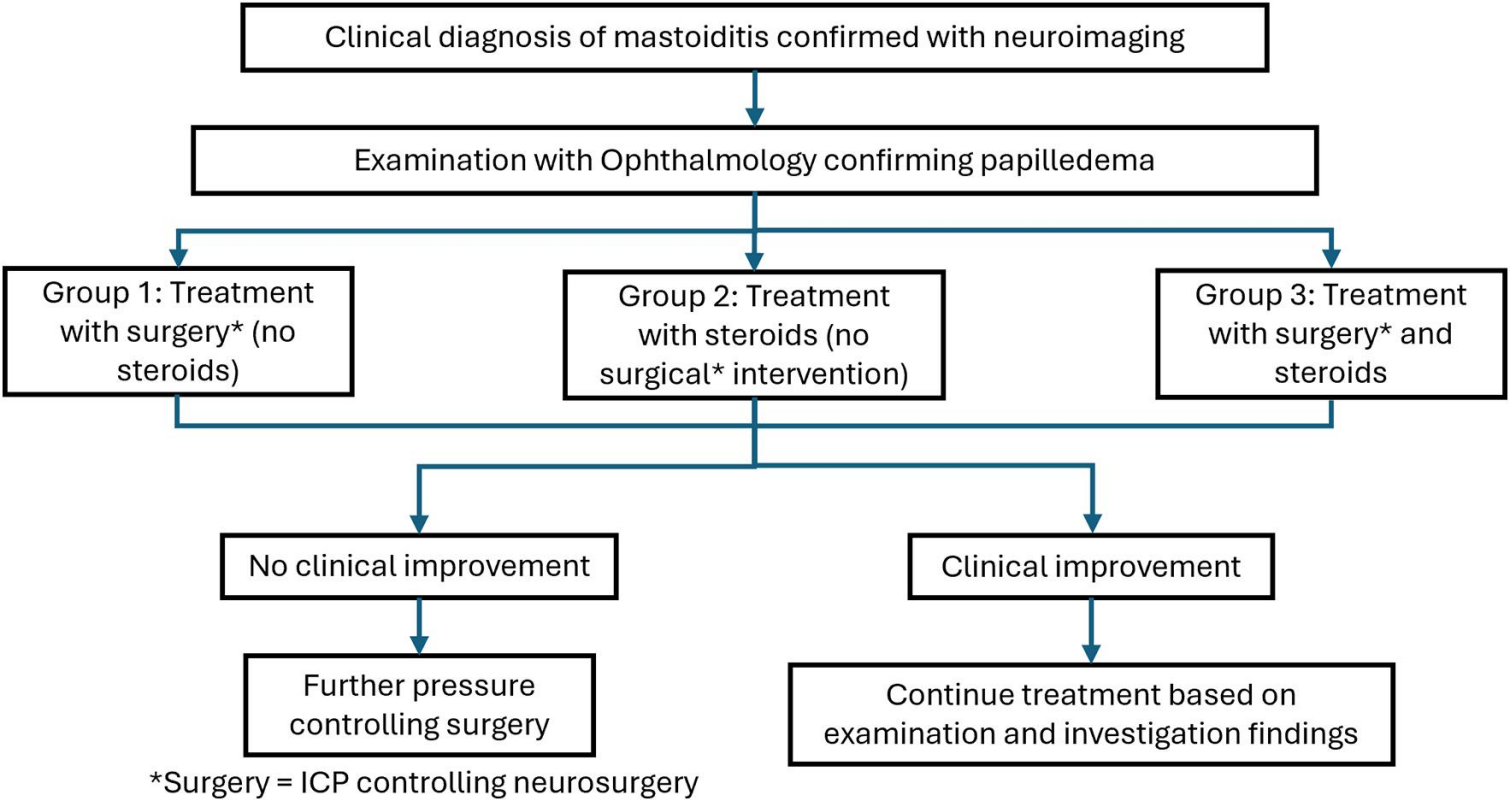
A flowchart illustrating the three different groups into which patients were divided based on their treatment.

Patients were split into three distinct groups based on the treatment received (Figure 1):

Group 1—Patients who had further ICP controlling neurosurgery and no corticosteroids (*n* = 2).
Group 2—Patients who had corticosteroids and no further ICP controlling neurosurgery (*n* = 8).
Group 3—Patients who had both corticosteroids and further ICP controlling neurosurgery (*n* = 4).

When comparing BCVA and papilledema grade between the three groups of patients, the worst VA and Frisen grade was used. The distinction between patients in Group 2 who had no further surgeries and those in Group 3 who had further ICP controlling surgery was dependent on the progression of papilledema. Patients in Group 3 demonstrated worsening papilledema despite corticosteroid, medical, and surgical treatment and therefore needed further neurosurgery to control ICP.

An analysis was carried out using IBM SPSS Statistics, Version 29.0.0.0 (IBM Corp., Armonk, NY, USA). The data were not normally distributed therefore we used non-parametric tools for the statistical analysis. The Kruskal–Wallis test was used to compare the three patient groups. The Wilcoxon rank-sum test was used to compare two groups (Group 2 vs. Group 3). The exact method was applied because of the small sample size. The alpha level (type I error) was set at 0.05.

## Results

A total of 14 patients were identified with otitic hydrocephalus. The mean age at presentation was 6 (range 2–9) years. Full demographic and treatment information is available in Table 1. Otalgia was the most common presenting complaint (86%), followed by fever (79%), headache (79%), diplopia/sixth cranial nerve palsy (71%), lethargy (64%). The average duration of symptoms before presentation was 11 days (range 2–29 days).

**Table 1:** An overview of the 14 patients in this case series.

Patient group and demographic				Initial ophthalmic examination					Interventions									Final ophthalmic examination				General outcomes
Case ID	Group	Gender	Age	BCVA in worse seeing eye	Worse Grade of Papilledema	CN Palsy	Unilateral or Bilateral Mastoiditis	OCT Retinal findings	Initial IV Antibiotic Treatment	Initial dose of steroids	Initial anticoagulant treatment	Starting dose of Acetazolamide (mg/kg)	Highest dose of Acetazolamide	Lumbar Puncture	Opening Pressure	Initial Surgery	Further ICP controlling neurosurgery	Papilledema Progression or improvement	OCT Retinal findings	Optic Disc Atrophy	Time to Resolution of Papilloedema (days)	Total duration of Hospital Admission (days)
1	1	F	5	20/40	Grade 2	Nil	Left sided	Normal	Piperacillin- tazobactam	–	LMWH	15	30	Yes	37	Left mastoidectomy with postauricular abscess drainage and tympanostomy tube placement	VP shunt	Progression	Normal	No	115	29
2	1	M	5	20/20	Grade 2	Nil	Bilateral	SRF + PAMM + IRF + exudate + PAMM	Vancomycin + Cefepime	–	Unfractionated Heparin	15	20	Yes	41	Bilateral Mastoidectomy and tympanostomy tube placement	VP shunt with certas valve and antisiphon	Progression	OS/IS dysruption	Yes	58	26
3	2	M	6	20/20	Grade 1	Nil	Right sided	PAMM	Ampicillin	20 mg/kg IV methyprednisolone	LMWH	15	30	Yes	27	Right mastoidectomy and bilateral tympanostomy tube placement	–	Progression	Normal	Yes	153	35
4	2	F	3	20/25	Grade 4	6th CN Palsy	Right sided	–	Ceftriaxone	25 mg/kg IV methylprednisolone	LMWH	15	25	No	–	Bilateral myringotomy with tympanostomy tube placement	–	Improvement	–	No	40	8
5	2	M	3	20/30	Grade 3	Nil	Right sided	–	Linezolid	1 mg/kg PO prednisolone	LMWH	15	20	No	–	–	–	Improvement	–	No	85	10
6	2	F	4	20/25	Grade 2	Nil	Right sided	Normal	Piperacillin- tazobactam	1 mg/kg PO prednisolone	Unfractionated Heparin	15	18	No	–	Right mastoidectomy, posterior auricular abscess drainage, and tympanostomy tube placement	–	Progression	Normal	No	83	16
7	2	M	8	20/20	Grade 2	6th CN Palsy	Right sided	Normal	Ceftriaxone + Metronidazole	20 mg/kg IV methlyprednisolone	LMWH	10	15	No	–	Epidural abscess drainage and bilateral myringotomy and tympanostomy tube placement	–	Improvement	Normal	No	68	17
8	2	F	2	F + F	Grade 2	6th CN Palsy	Left sided	–	Cephtriaxone	25 mg/kg IV methylprednisolone	LMWH	15	25	No	–	Bilateral myringotomy with tympanostomy tube placement	–	Improvement	–	No	45	7
9	2	F	8	20/20	Grade 4	6th CN Palsy	Right sided	SRF + IRF	Ceftriaxone + Metronidazole	30 mg/kg IV methylprednisolone	LMWH	15	20	No	–	Right tympanostomy tube placement	–	Improvement	PAMM + exudates	Yes	144	12
10	2	M	9	20/20	Grade 1	Nil	Right sided	Normal	Ceftriaxone + Metronidazole + Linezolid	1 mg/kg IV Dexamethasone	Unfractionated Heparin	10	20	No	–	Bilateral myringotomy with tympanostomy tube placement	–	Progression	Normal	No	21	14
11	3	M	5	20/40	Grade 1	6th CN Palsy	Left sided	Normal	Ceftriaxone + Metronidazole + Vancomycin	30 mg/kg IV methylprednisolone	LMWH	15	–	Yes	30	Left mastoidectomy and bilateral tympanostomy tube placement	Lumbar Drain	Progression	Normal	No	155	24
12	3	M	5	20/30	Grade 3	Nil	Left sided	Grade 1 Foveal Hypoplasia	Meropenem + Vancomycin	30 mg/kg IV methylprednisolone	Unfractionated Heparin	20	25	Yes	55	Myringotomy with tympanostomy tube placement	VP shunt	Progression	Normal	No	131	18
13	3	M	7	20/25	Grade 3	Nil	Right sided	SRF	Ceftriaxone + Metronidazole + Vancomycin	0.5 mg/kg IV dexamethasone	LMWH	15	–	No	–	Bilateral myringotomy with tympanostomy tube placement	Bilateral cerebral thrombectomy and angioplasty and left cerebral stent placement	Progression	Normal	Yes	48	25
14	3	M	8	20/25	Grade 4	6th CN Palsy	Left sided	SRF + IS/OS disruption	Ceftriaxone + Metronidazole + Linezolid	30 mg/kg IV methylprednisolone	LMWH	20	–	No	–	Left mastoidectomy and perisigmoid abscess drainage	Cerebral angiography and venous mechanical thrombectomy	Improvement	IS/OS disruption	Yes	38	16

The table highlights the examination findings and the interventions received.

BCVA, best-corrected visual acuity; LMWH, low-molecular-weight heparin; OCT, optical coherence tomography; IV, intravenous; SRF, subretinal fluid; IRF, intraretinal fluid; PAMM, paracentral acute middle maculopathy; IS/OS, inner segment and outer segment junction; CN, cranial nerve; F + F, fix and follow; F, female; M, male; VP, ventriculoperitoneal.

**Figure 2:**
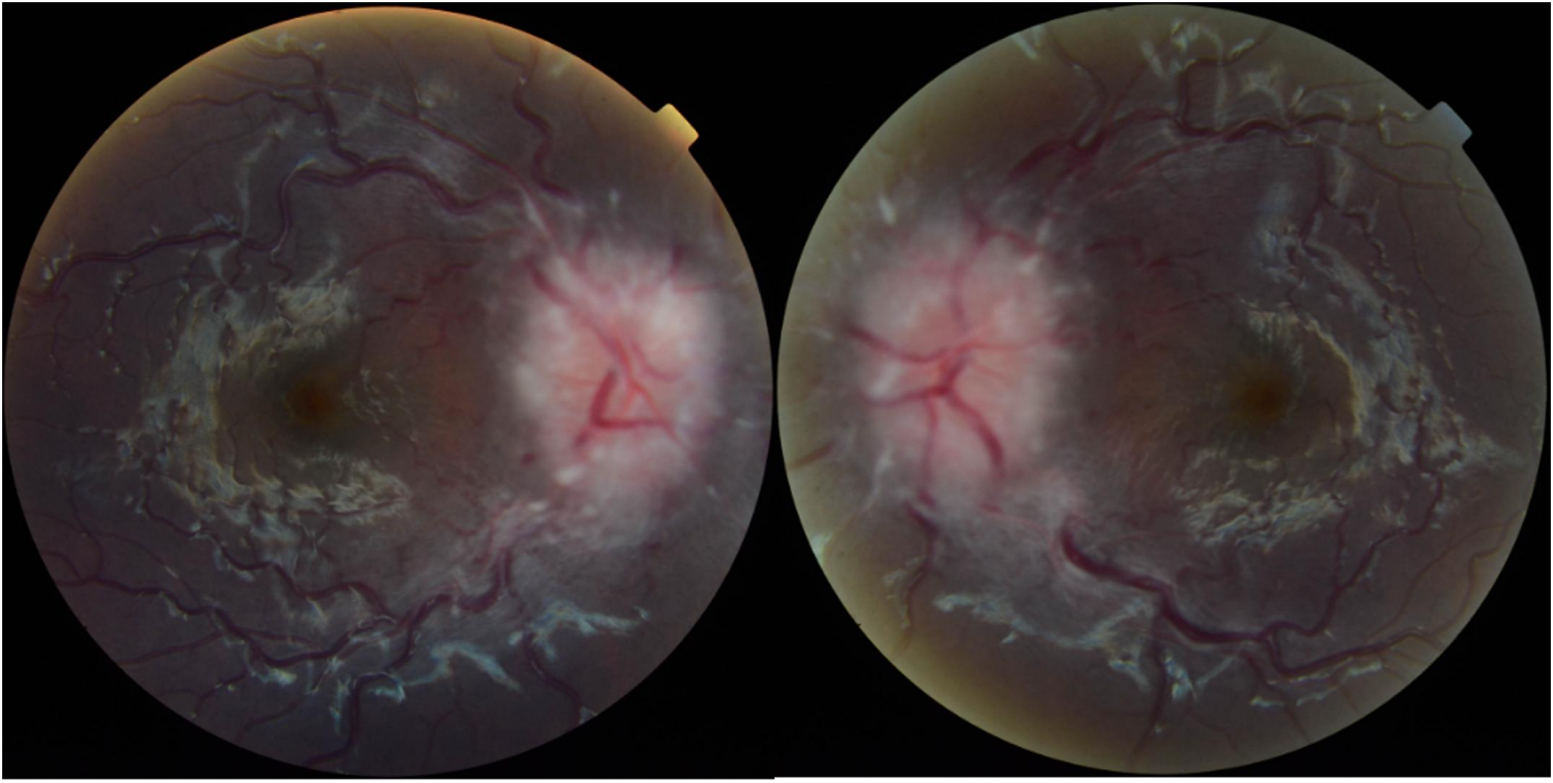
A topcon fundus photograph of a patient presenting grade 4 papilledema caused by otitic hydrocephalus.

**Figure 3:**
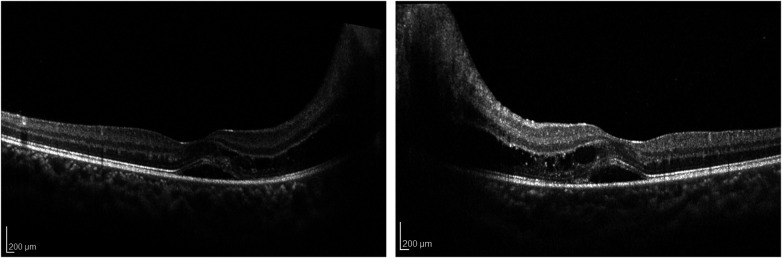
A Heidelberg OCT showing subretinal fluid, intraretinal fluid, and retinal exudate as a result of severe papilledema.

At presentation, patients' BCVA in the worse seeing eye ranged from 20/20 to 20/40, except one who was only able to fix and follow. Optic disc edema at presentation ranged from Frisen grade 1 to grade 4 (Figure 2), with eight (57%) patients presenting with grade 1 or 2 papilledema (Table 2). Six patients (43%) had cranial nerve VI palsy. A total of 11 patients underwent an OCT examination on presentation (3 were not developmentally able), and the results revealed an average RNFL thickness of 262 µm and an average GCL volume of 1.1 mm^3^) in the worse eye. Five patients had abnormal retinal findings such as subretinal fluid (SRF), intraretinal fluid (IRF), retinal exudates, inner segment and outer segment junction (IS/OS) disruption, and paracentral acute middle maculopathy (PAMM) (Figure 3).

**Table 2:** Demographics and optical coherence tomography findings for patients with otitic hydrocephalus.

Demographics	Total patients (% of total)	Group 1 (% of group 1)	Group 2 (% of group 2)	Group 3 (% of group 3)
No. of patients	14	2	8	4
Male	9 (64.3%)	1 (50%)	4 (50%)	4 (100%)
Female	5 (35.7%)	1 (50%)	4 (50%)	0 (0%)
Average age (years)	5.6	5.0	5.4	6.3
Initial ophthalmology exam				
Frisen Grade 1	3 (21.4%)	0 (0%)	2 (25%)	1 (25%)
Frisen Grade 2	6 (42.9%)	2 (100%)	3 (75%)	0 (0%)
Frisen Grade 3	2 (14.3%)	0 (0%)	1 (12.5%)	2 (50%)
Frisen Grade 4	3 (21.4%)	0 (0%)	2 (25%)	1 (25%)
Cranial Nerve VI palsy	6 (42.9%)	0 (0%)	4 (50%)	2 (50%)
Average GCL volume	1.11	1.03	1.10	1.15
Average RNFL thickness	262.4	265.8	207.0	316.0
Final ophthalmology examination				
Average GCL volume	1.02	0.93	1.07	1.02
Average RNFL thickness	106.3	100.3	112.8	101.1
Atrophy on final examination	5 (35.7%)	1 (50%)	2 (25%)	2 (50%)
Retinal findings on final examination	5 (35.7%)	1 (50%)	2 (25%)	2 (50%)

GCL, ganglion cell layer; RNFL, retinal nerve fiber layer.

CT and MR examinations were reviewed for 13 of 14 patients; the reports were reviewed for 1 patient imaged at an outside institution, for whom images were not available. The CT of the temporal bones was obtained in 13 of 14 patients, and MR with MRV was obtained in all patients, with 12 MR images obtained on a 3T system at our institution. CVST was present in all patients and involved a part or most of one transverse sinus (TVS) (*n* = 13) extending to the contralateral TVS in one of these patients, one sigmoid sinus (SS) (*n* = 14), and one internal jugular vein (IJV) (*n* = 12). CVST involved the dominant TVS and SS in seven patients, with the remaining seven patients having codominant (*n* = 5), nearly symmetric sinuses (*n* = 2). Papilledema was present in all 13 patients for whom MR was available for review as evidenced by a protrusion of the optic papillae on T2-weighted axial images. Ipsilateral perisinus epidural abscesses were present in 8 patients and bilateral abscess in 1. Mastoid and middle ear space fluid signal was present ipsilateral to CVST in all patients and was bilateral in 11 patients. All 13 patients imaged with CT had evidence of coalescent mastoiditis with a mastoid air cell and middle ear space opacification and demineralization or erosion of the mastoid cortex.

All patients were started on broad-spectrum intravenous antibiotics (IV) within one day of admission. All were anticoagulated. Acetazolamide was started after the first ophthalmic examination, at 10–20 mg/kg/day, and in some patients, it was increased up to 30 mg/kg/day. Corticosteroids were started for 12 patients after controlling infection, with the average time to corticosteroids of 10 days (range 0–30 days). The patients were started on either 20–30 mg/kg IV methylprednisolone, 0.50–1 mg/kg dexamethasone, or 0.75–2 mg/kg oral prednisolone.

Thirteen patients were treated with initial otologic surgery with the aim of primary source control. These surgeries included canal wall up mastoidectomy, tympanostomy tube insertion, and drainage of postauricular abscess.

LP was performed for five patients as part of their diagnostic workup or for evaluation of refractory symptoms and to guide further management. The average opening pressure was 38 cm H_2_O (range 27–55). Most of the time, LP was performed for refractory cases (*n* = 4).

### Group 1: patients who had further ICP controlling neurosurgery and no corticosteroids

When reviewing two patients in Group 1, the initial otologic surgeries included canal wall up mastoidectomy and myringotomy with tympanostomy tube placement. Both patients showed signs of worsening papilledema, and therefore, further neurosurgical intervention was required to control ICP. Both underwent attempted mechanical thrombectomy that was unsuccessful and subsequent ventriculostomy catheter placement. The time to resolution of papilledema was 58 and 115 days. Fortunately, there were no permanent VA changes; however, one patient demonstrated photoreceptor damage (i.e., IS/OS disruption) and optic disc atrophy following resolution of papilledema on OCT. This patient remains too young for undergoing formal automated perimetry; however, a central visual field deficit related to the disruption of the photoreceptors in his macula is suspected.

### Group 2: patients who had corticosteroids and no further ICP controlling neurosurgery (*n* = 8)

Eight patients in Group 2 had additional corticosteroids to help manage increased ICP. The average time to corticosteroid initiation was 12 days and a majority of them showed signs of improvement in the condition of papilledema. Three patients (38%) had a progression of papilledema during their treatment course. In the patients who showed signs of progression, the corticosteroids were started one day earlier or after the documentation of progression. Upon starting the drugs, the condition of papilledema had improved at their next appointment. The average time to resolution of papilledema was 80 days. On final examination, the patients had normal VA and OCT; however, one patient (12.5%) had disc atrophy, and another patient had signs of PAMM and macular exudates.

### Group 3: patients who had both corticosteroids and further ICP controlling neurosurgery (*n* = 4)

Four patients in Group 3 were given corticosteroids and underwent otologic surgery as part of their initial management, much like Group 2. Unfortunately, these four patients had worsening papilledema on ophthalmic examination. Therefore, they required neurosurgical intervention, including lumbar drain, ventriculostomy catheter and cerebral endovascular thrombectomy, angioplasty, and stent placement. The average time to corticosteroids was 5 days and the average time to further ICP controlling surgery was 20 days. The time to resolution of papilledema was 93 days. No permanent visual acuity changes were noted on final examination; however, 50% of patients had optic disc atrophy.

Five patients (36%) across the three groups developed bilateral optic disc atrophy (median 58 days, interquartile range 48–144). After surgery, papilledema progressed in three of these patients with an average time of progression of 15 days after surgery. In the other two patients, the condition of papilledema improved at the next ophthalmic review. The opening pressure was recorded at 27 and 41 cm H_2_O for the two patients. All patients had macular OCT signs on initial examination, and the most common finding was subretinal fluid, which was present in four patients. The other notable findings were PAMM (*n* = 2), IR (*n* = 2), and OS/IS disruption (*n* = 1). All patients were treated with corticosteroids. Four patients underwent initial otologic surgeries and three patients required further ICP control with neurosurgical procedures. On final examination, three patients still had abnormal OCT retinal findings, including two patients with IS/OS disruption and one patient with PAMM and exudates. The average VA was 20/24 in all these patients. The average RNFL and GCL were 104 µm and 1.01 mm^3^, respectively.

On average, patients were admitted to hospital for 18 days [standard deviation (SD) 8.34, range 7–25] days. When comparing the three groups, it was found that there was a significant difference in the duration (*p* = 0.05). Patients in Group 1 were admitted to hospital for an average of 27 (SD 2.12, range 26–29) days. Patients in Group 2 were admitted to hospital for an average of 15 (SD 8.89, range 7–35) days. Finally, patients in Group 3 were admitted to hospital for an average of 21 (SD 4.43, range 16–25) days.

Patients in Group 3 required further surgery because of an intractable progression of papilledema; they were, therefore, deemed as patients in whom corticosteroid treatment had failed. We evaluated the possible risk factors associated with corticosteroid treatment failure. When comparing worse seeing BCVA in Group 2 (20/23, SD 3.93, range 20/20–20/30) and Group 3 patients (20/30, SD 7.07, range 20/25–20/40), no significant difference (*p* = 0.10) was found. There was no significant difference between the grade of papilledema between Group 2 and Group 3 patients (*p* = 0.79). None of the blood inflammatory markers, including peak ESR, CRP, procalcitonin, or WBC, were a significant risk factor for corticosteroid failure (Table 3).

**Table 3:** A statistical analysis of risk factors to understand the difference between steroid responders and non-responders.

Parameters	Total	Failed steroid treatment or not		*p*-Value
		Steroids and surgery^a^ (Group 3)	Steroids only (Group 2)	
Worse VA				
*N*	*N* = 11	*N* = 4	*N* = 7	0.10
Mean (SD)	25.5 (6.11)	30 (7.07)	22.9 (3.93)	
Median (IQR)	25 (20, 30)	27.5 (25, 35)	20 (20, 25)	
(Min, Max)	(20, 40)	(25, 40)	(20, 30)	
Worse Grade of Papilledema				
*N*	*N* = 12	*N* = 4	*N* = 8	0.79
Median (IQR)	2.5 (1.5, 3.5)	3 (2, 3.5)	2 (1.5, 3.5)	
(Min, Max)	(1, 4)	(1, 4)	(1, 4)	
Peak ESR				
*N*	*N* = 9	*N* = 4	*N* = 5	>0.99
Mean (SD)	57.1 (41.37)	56.3 (39.93)	57.8 (47.19)	
Median (IQR)	79 (11, 87)	60.5 (23.5, 89)	79 (11, 87)	
(Min, Max)	(4, 108)	(9, 95)	(4, 108)	
Peak CRP				
* N*	*N* = 12	*N* = 4	*N* = 8	0.46
Mean (SD)	23.9 (35.88)	26.1 (21.99)	22.8 (42.56)	
Median (IQR)	8.6 (0.8, 32.4)	25.2 (12.5, 39.8)	1.9 (0.7, 27.2)	
(Min, Max)	(0.06, 122.7)	(0.2, 54)	(0.06, 122.7)	
Peak WBC				
*N*	*N* = 12	*N* = 4	*N* = 8	0.57
Mean (SD)	18.2 (5.51)	20.2 (3.22)	17.1 (6.30)	
Median (IQR)	17.7 (13.9, 22.9)	19.7 (17.5, 22.9)	17.2 (11.1, 22.3)	
(Min, Max)	(9.5, 26.3)	(17.5, 24)	(9.5, 26.3)	
Peak Procalcitonin				
*N*	*N* = 5	*N* = 1	*N* = 4	N/A
Mean (SD)	20.1 (36.73)	84.9 (.)	3.9 (7.18)	
Median (IQR)	1 (0.07, 14.7)	84.9 (84.9, 84.9)	0.5 (0.05, 7.9)	
(Min, Max)	(0.03, 84.9)	(84.9, 84.9)	(0.03, 14.7)	
Time to steroids				
*N*	*N* = 12	*N* = 4	*N* = 8	0.49
Mean (SD)	9.4 (9.33)	7 (9.20)	10.6 (9.77)	
Median (IQR)	7 (1, 16)	4 (0.5, 13.5)	7.5 (3.5, 16)	
(Min, Max)	(0, 30)	(0, 20)	(1, 30)	

The Wilcoxon rank-sum test was used to compare two groups of patients. The exact method was applied because of the small sample size.

^a^ Further ICP controlling neurosurgery.

The dominant TVS, SS, and IJV were involved in three of four patients in Group 3, with the most longitudinally extensive CVST seen in one of these patients. In Groups 1 and 2, there was an involvement of the dominant TVS, SS, and IJV in four of 10 patients.

## Discussion

In our evaluation of the impact of corticosteroids on clinical outcomes, we found that patients who were treated with corticosteroids for otitic hydrocephalus had an overall reduction in hospital stay. When comparing our three groups, we found no significant difference between the presenting symptoms and examination findings between them, suggesting that it could be difficult to predict which patients would require further surgeries despite intravenous corticosteroids. However, involvement of the dominant TVS, SS and IJV was seen more frequently in Group 3 patients.

A previous study by Chen et al., noted the potential benefit of using systemic corticosteroids in patients who were poorly responsive to medical management (15). They hypothesized that the worsening of papilledema was due to inflammation occurring locally. In patients with acute pseudotumor cerebri, high-dose corticosteroids were found to be effective in managing acute vision loss (18). In our study, 66% of those who were treated with corticosteroids did not require further neurosurgery.

Antibiotics and otologic surgical intervention are part of established management for intracranial hypertension secondary to mastoiditis (3, 10, 12, 19) While these work well to control the primary disease process, increased ICP and threat to vision remain a serious sequelae. The main morbidity resulting from them is headache and potential vision loss, and while the visual acuity outcomes of all 14 patients were good, there was evidence of optic nerve atrophy in 35.7% (5/14) of patients. All patients in our series received anticoagulation, and despite this, half the patients required neurosurgical intervention as there was refractory intracranial hypertension despite treatment of the infection and thrombus (20). Our group previously made similar observations and hypothesized that a secondary inflammatory process, following the initial infection, may cause dysregulation of the cerebrospinal fluid cycle; this has been noted in other studies of CVST without infection as well (6, 15). This phenomenon has been described to occur in 21.5%–29% of patients with CVST from all causes (4, 14).

In the cohort of patients who had corticosteroids only, 33% went on to require further neurosurgical intervention for vision-threatening papilledema. Cerebrospinal fluid shunting (ventriculostomy catheter, lumboperitoneal shunt, or lumbar drain) is a traditional option for treatment for persistently increased ICP. Previous studies have shown that these interventions can be effective in reducing ICP (13). As fewer patients in our study required surgery following systemic corticosteroids, we suggest that including steroids as part of treatment may reduce the need for neurosurgical intervention.

We performed a subanalysis, only looking at patients who had corticosteroids vs. those who went on to require further neurosurgery. We evaluated whether there were any differences between VA and papilledema grade on presentation. We found that there was no significance between the two groups; however, VA was trending toward significance (*p* = 0.10). This could be investigated further with a larger cohort of patients. Patients receiving corticosteroids had reduced hospital length of stay in between the three groups of patients (*p* = 0.05). This may indicate that there is a role for corticosteroids in patients with mastoiditis complicated by papilledema and CVST as they can help reduce psychological and physical burden on children and their families by reducing their length of stay. They may also serve as an indirect surrogate marker of overall systemic benefit in these patients.

Within the subcohort of patients who had retinal abnormalities on presentation (*n* = 5), all patients went on to develop optic disc atrophy after resolution of their papilledema. Fortunately, in these patients, final VA returned to normal despite OCT findings. Previous literature suggests that there can be a discrepancy between VA and optic disc atrophy; however, there may be a higher risk of future visual limitations (21). Furthermore, given the developmental age of the patients in our cohort, it was sometimes difficult to obtain reliable visual field assessments that may have demonstrated visual field loss. This emphasizes the importance of closer follow-up and more aggressive intervention. Curley et al. found that patients with retinal manifestations in idiopathic interstitial hypertension, such as retinal fluid, were more likely to have worse visual function and outcomes, indicating that macular findings at presentation may be a factor for poor prognosis (22). Therefore, in addition to monitoring patients who demonstrate signs of optic disc atrophy, a close evaluation of patients with retinal changes at presentation is warranted. OCT retinal imaging in patients with a similar clinical presentation may represent a useful tool for risk stratification. Prospective studies can help determine whether retinal changes on OCT can predict disease progression.

### Limitations

This study has several limitations. First, this is a retrospective review of a fairly rare condition (15) and therefore the sample size is limited in this study. The smaller sample size means that causality cannot be inferred because of confounding factors such as severity of disease, time to treatment, and involvement of dominant sinus. Further studies with larger sample sizes are required to investigate the casual relationship between the use of corticosteroids and improvement in patient visual outcome. Second, the treatment of this condition at our institution evolved from the initial cohort of patients whom we treated to the current cohort, and the data reflect our changing treatments in response to therapeutic efficacy. As a result, there is heterogeneity in corticosteroid type between patients depending on clinical presentation and severity of symptoms. Therefore, some amount of caution should be exercised when deriving conclusions on how corticosteroid treatment can impact the progression of disease.

## Conclusion

In conclusion, our series shows that there could be a potential role for systemic corticosteroids in reducing the hospital duration of patients with mastoiditis complicated by CVST and increased ICP. When patients are started on corticosteroids, it can be difficult to predict which patients may require further neurosurgery to control ICP. Therefore, it is important to monitor patients closely for improvement in the condition of papilledema.

Patients who presented with retinal changes on OCT may be at a higher risk of developing optic disc atrophy, which, in turn, may impact visual function such as the visual field. Thus, such patients require closer monitoring and more aggressive therapy.

## Data Availability

The original contributions presented in the study are included in the article/Supplementary Material, and further inquiries can be directed to the corresponding author.
